# Data on the application of Functional Data Analysis in food fermentations

**DOI:** 10.1016/j.dib.2016.09.013

**Published:** 2016-09-15

**Authors:** M.A. Ruiz-Bellido, V. Romero-Gil, P. García-García, F. Rodríguez-Gómez, F.N. Arroyo-López, A. Garrido-Fernández

**Affiliations:** a Regulatory Council of PDO Aloreña de Málaga Table Olives, C/Dehesa, 80, 29560 Pizarra, Málaga, Spain; b Food Biotechnology Department, Instituto de la Grasa (IG-CSIC), University Campus Pablo de Olavide, Building 46, Ctra, Utrera, km 1, 41013 Seville, Spain

## Abstract

This article refers to the paper “*Assessment of table olive fermentation by functional data analysis”* (Ruiz-Bellido et al., 2016) [1]. The dataset include pH, titratable acidity, yeast count and area values obtained during fermentation process (380 days) of *Aloreña de Málaga* olives subjected to five different fermentation systems: i) control of acidified cured olives, ii) highly acidified cured olives, iii) intermediate acidified cured olives, iv) control of traditional cracked olives, and v) traditional olives cracked after 72 h of exposure to air. Many of the Tables and Figures shown in this paper were deduced after application of Functional Data Analysis to raw data using a routine executed under R software for comparison among treatments by the transformation of raw data into smooth curves and the application of a new battery of statistical tools (functional pointwise estimation of the averages and standard deviations, maximum, minimum, first and second derivatives, functional regression, and functional *F* and *t*-tests).

**Specifications Table**

**Table:** 

Subject area	*Food Technology*
More specific subject area	*Microbiology, Statistics*
Type of data	*Tables, figures*
How data was acquired	*Use of a titroprocessor mod 670 (Metrohm Instrument, Herisau, Switzerland) for determination of pH and titratable acidity values. Use of a Spiral System model dwScientific (Dow Whitley Scientific Limited, England) for determination of yeast counts on selective medium.*
Data format	*Raw, analyzed*
Experimental factors	*Five fermentation systems of cured and cracked Aloreña olives with different NaCl and acidification conditions.*
Experimental features	*Monitoring of fermentations, microbial and physicochemical analysis, transformation of data into smooth curves, functional data analysis*
Data source location	*Alozaina, Málaga, Spain.*
Data accessibility	*Data available within this article*

**Value of the data**

• Use datasets as a benchmark for further functional data analysis or modelling of table olive fermentations.
• Application of functional data analysis for the study of food fermentations.
• Understand the influence of acidification and cracking of olives on the fermentation process of *Aloreña olives* by comparisons among different fermentation systems.

## 1 Data

**Fig. 1:**
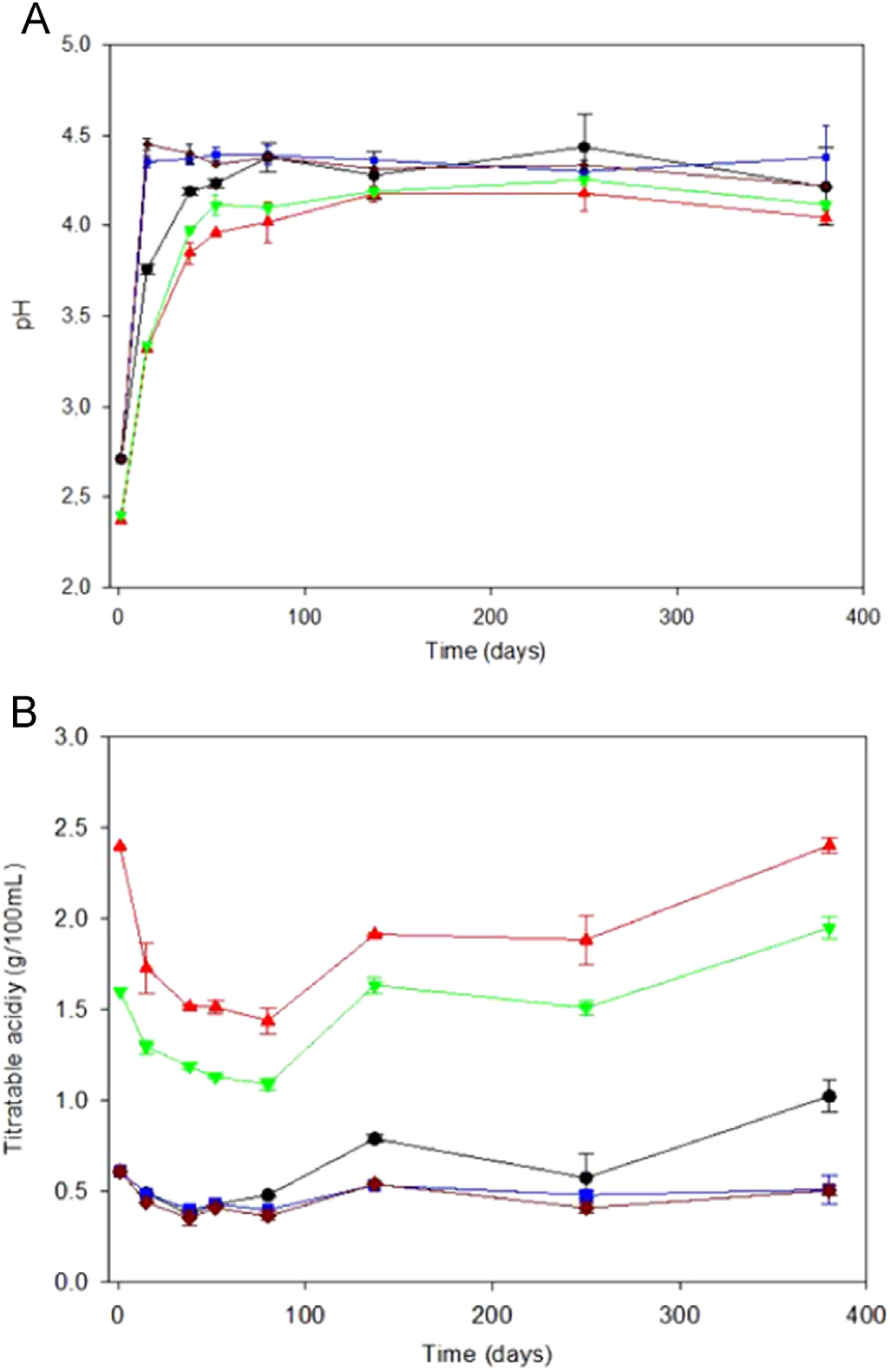
Changes in pH (panel A) and titratable acidity (panel B) over time, according to treatments ( and ). CC, control of acidified cured olives; CI, highly acidified cured olives; CII, intermediate acidified cured olives; CT, control of traditional (cracked) olives; RT, traditional olives, cracked after 72 h of exposure to air.

**Fig. 2:**
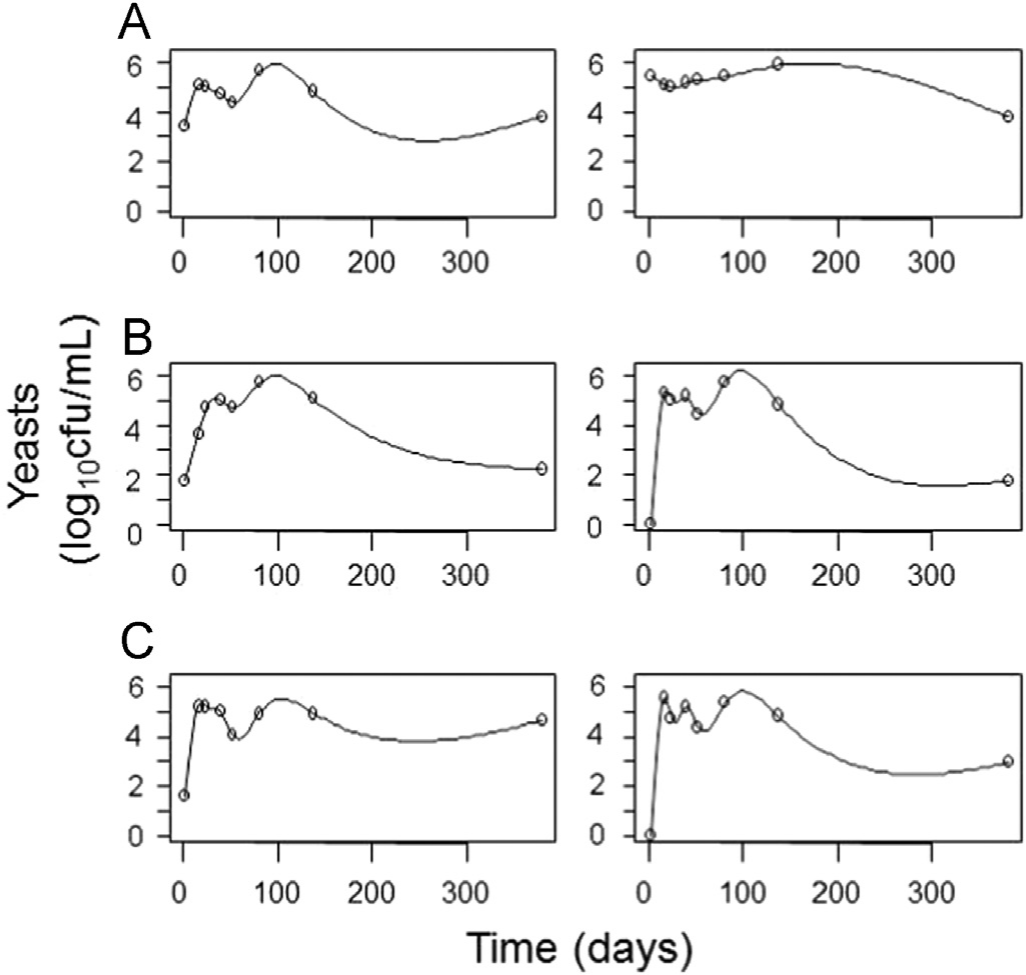
Graphical presentation of some examples of yeast population smoothing; each row shows the two replicate of treatments CC (panel A), CI (panel B) and CII (panel C). CC, control of acidified cured olives; CI, highly acidified cured olives; CII, intermediate acidified cured olives.

**Fig. 3:**
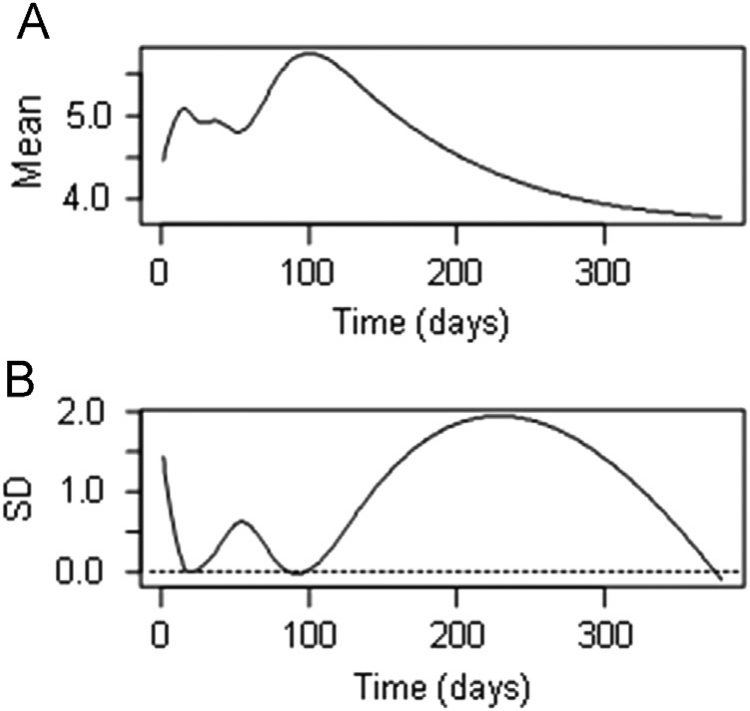
Estimations of the average mean (panel A) and standard deviation (panel B) yeast in treatment CC, expressed as log_10_ cfu/ml, based on the yeast functional object obtained from smoothing. CC, control of acidified cured olives.

**Fig. 4:**
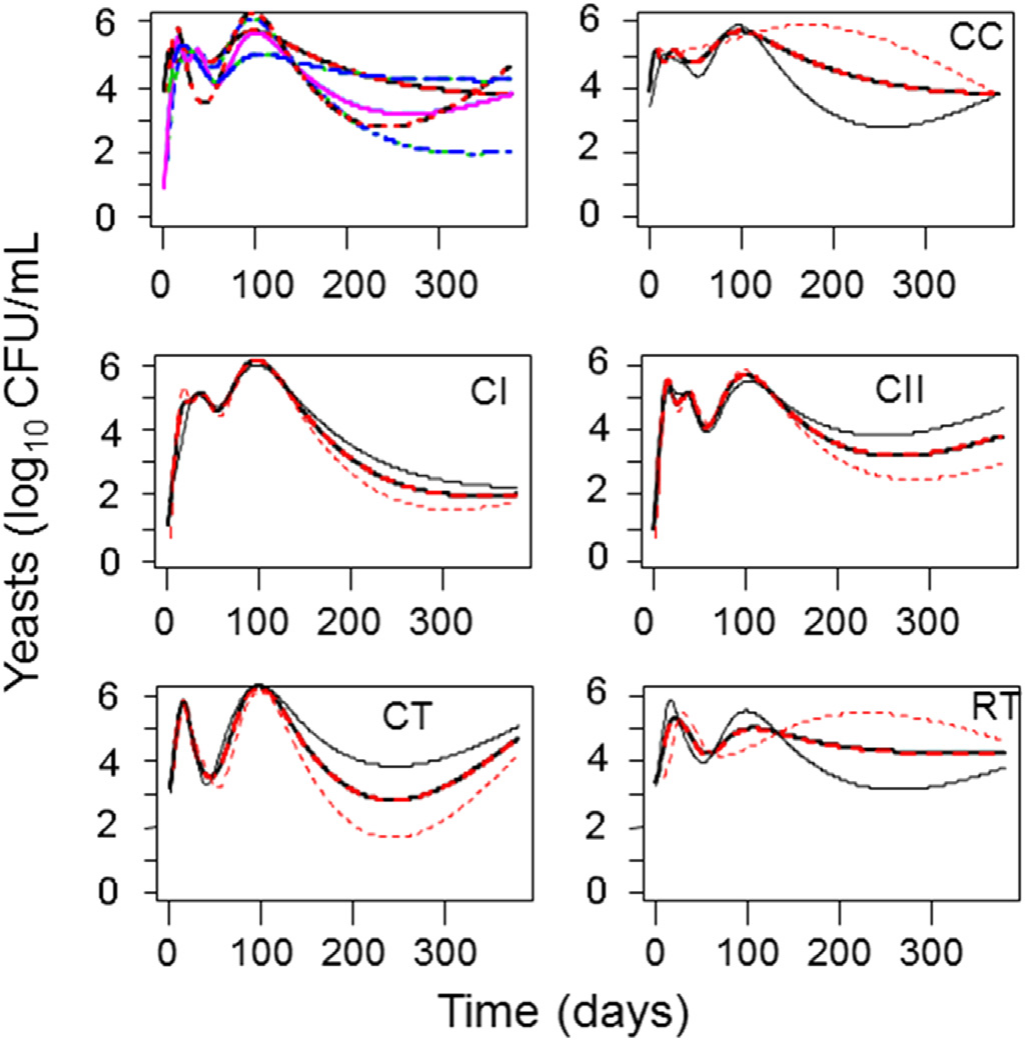
Functional regression, showing the overall trends obtained for all treatments assayed (top left), followed by the average (and their replicates) of the specific profiles for each of the treatments. CC, control of acidified cured olives; CI, highly acidified cured olives; CII, intermediate acidified cured olives; CT, control of traditional (cracked) olives; RT, traditional olives, cracked after 72 h of exposure to air.

**Fig. 5:**
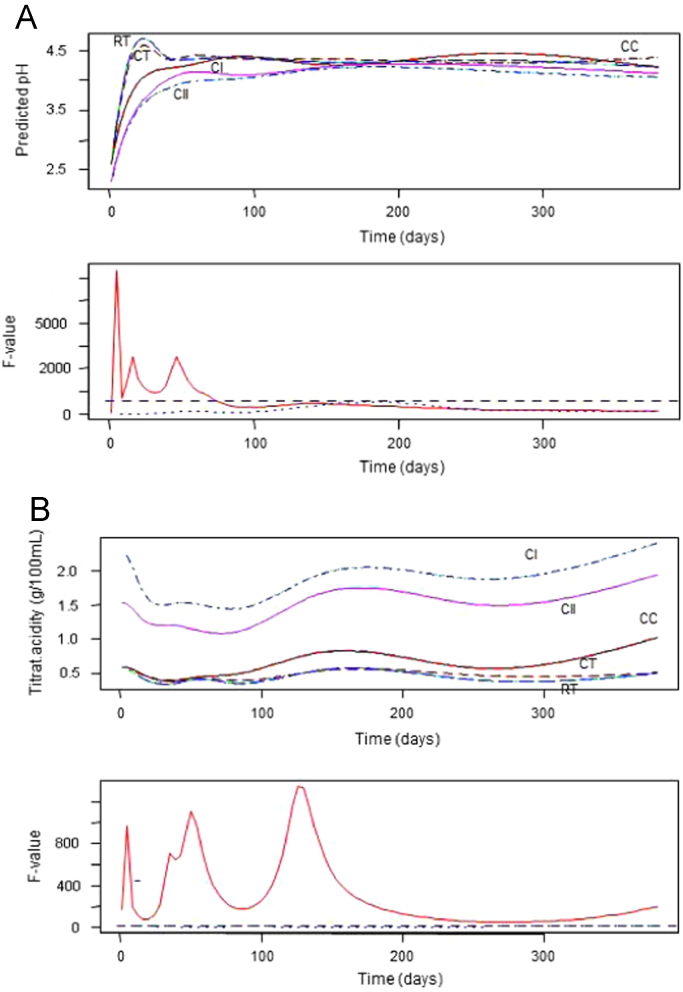
Functional analysis of variance for the changes in pH (panel A) and titratable acidity (panel B) over time. Panel A: upper graph, predicted pH regression curves for the treatments assayed; bottom graph, pH permutation *F*-test for the curves above. Panel B: upper graph, regression predicted titratable acidity curves for the treatments assayed; bottom graph, permutation *F*-test for the above curves. In both permutation tests, the graphs show the observed *F*-value, together with its maximum (break line) and pointwise 0.05 critical values (dotted lines). CC, control of acidified cured olives; CI, highly acidified cured olives; CII, intermediate acidified cured olives; CT, control of traditional (cracked) olives; RT, traditional olives, cracked after 72 h of exposure to air.

**Fig. 6:**
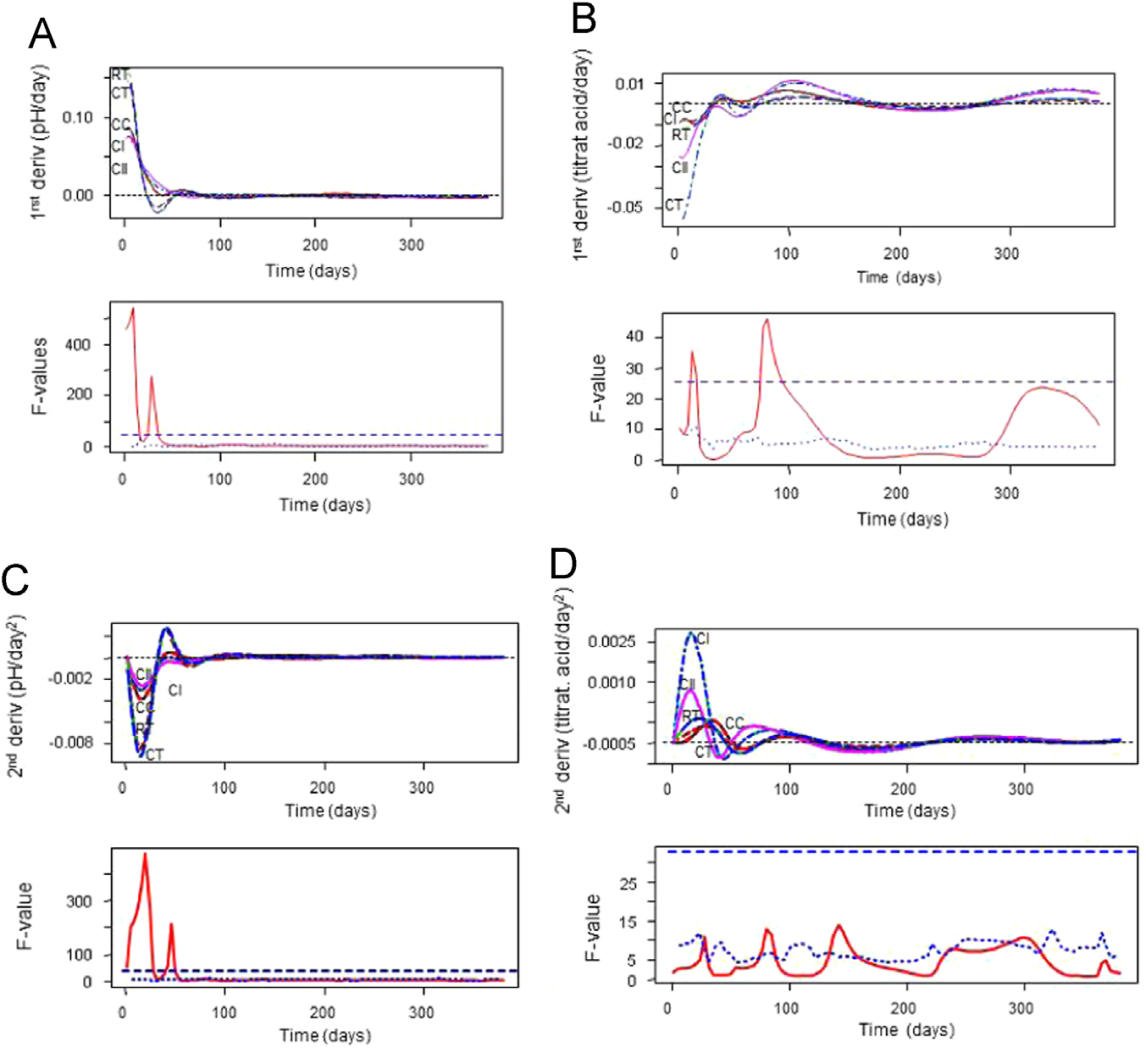
Functional analysis of variance for first (pH, panel A upper graph; titratable acidity, panel B upper graph) and second derivatives (pH, panel C upper graph; titratable acidity, panel D upper graph), and their respective estimated permutation functional *F*-tests (bottom curves of panels). For the *F*-test, the pointwise *F*-values, together with its maximum (broken lines) and pointwise (dotted line) *p*=0.05 critical values are indicated. CC, control of acidified cured olives; CI, highly acidified cured olives; CII, intermediate acidified cured olives; CT, control of traditional (cracked) olives; RT, traditional olives, cracked after 72 h of exposure to air.

**Fig. 7:**
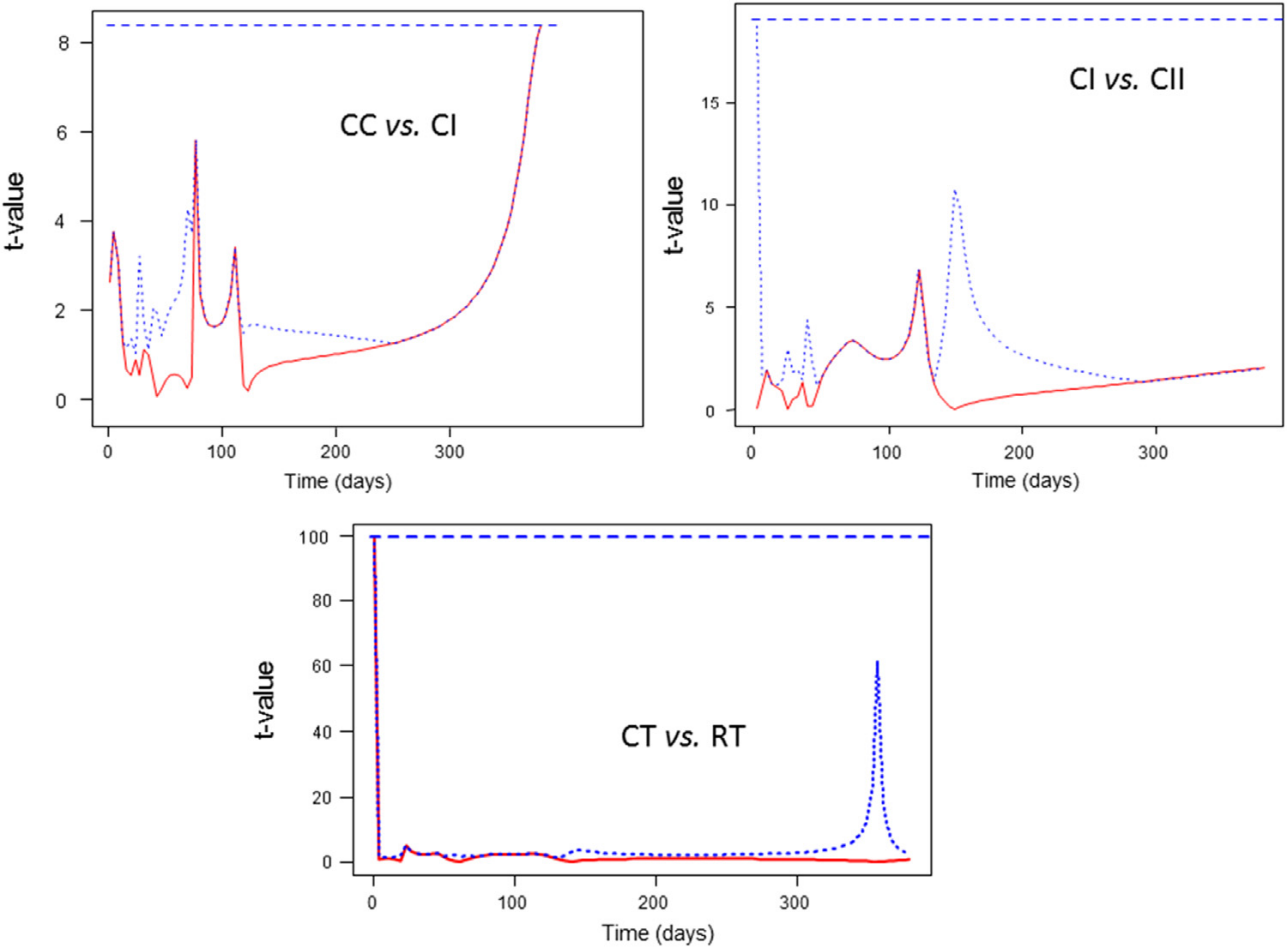
Functional permutation *t*-test for the comparison of yeast growth curves (CC vs. CI, CI vs. CII, and CT vs*.* RT). Graphs show the pointwise estimated *t*-test values together with their maxima (broken lines), and pointwise (dotted line) *p*=0.05 critical values. CC, control of acidified cured olives; CI, highly acidified cured olives; CII, intermediate acidified cured olives; CT, control of traditional (cracked) olives; RT, traditional olives, cracked after 72 h of exposure to air.

**Fig. 8:**
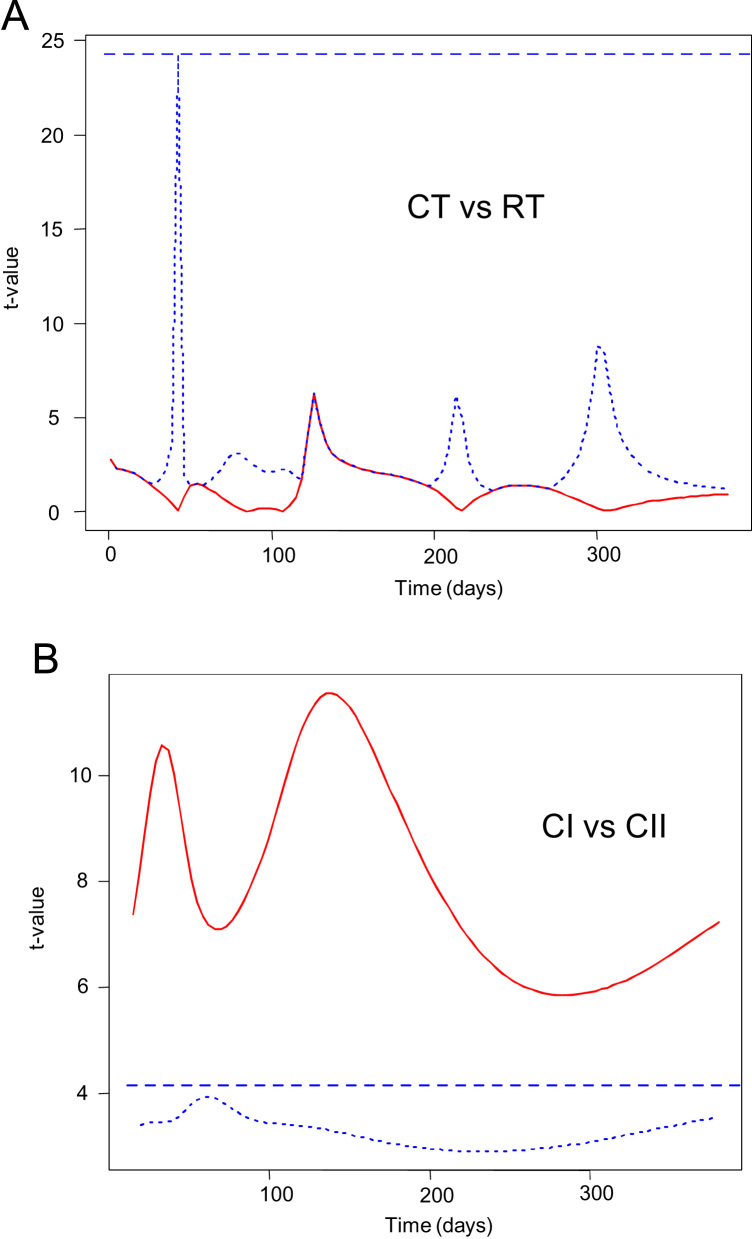
Permutation functional *t*-test for the comparison of pH changes in CT vs. RT (panel A) and titratable acidity changes in CI vs. CII (panel B). The graphs show the pointwise *F*-values, together with its maximum (broken lines) and pointwise (dotted line) *p*=0.05 critical values. CI, highly acidified cured olives; CII, intermediate acidified cured olives; CT, control of traditional (cracked) olives; RT, traditional olives, cracked after 72 h of exposure to air.

The dataset provided in this article (Table 1, Table 2, Table 3, Table 4, Table 5, Table 6, Table 7) and their corresponding Figures (Fig. 1, Fig. 2, Fig. 3, Fig. 4, Fig. 5, Fig. 6, Fig. 7, Fig. 8) represent the raw microbiological (yeast counts) and physicochemical (pH and titratable acidity) data, as well as their statistical analysis by the application and implementation of Functional Data analysis, of different olive fermentation systems using *Aloreña de Málaga fruits*.

## 2 Experimental design, materials and methods

Olives were harvested at the green ripe stage during the 2013/14 season (Valle del Guadalhorce, Málaga, Spain) and subjected to five different fermentation system: i) CC (usual brine, control cured olives): 7 g/100 ml NaCl, 0.1 g/100 ml citric acid (CA), 0.5 g/100 ml acetic acid (AA); ii) CI (highly acidified, cured olives): no salt, 0.1 g/100 ml CA, 1.6 g/100 ml AA; iii) CII (moderately acidified, cured olives): no salt, 0.1 g/100 ml CA, 1.0 g/100 ml AA; iv) CT (usual brine of cracked, traditional olives): 11 g/100 ml NaCl solution, and v) RT (usual brine, olives cracked after 72 h respiration at room temperature): brined in a 11 g/100 ml NaCl solution. For the rest of the details of the experimental design, and how microbiological and physicochemical data were acquired, please consult the paper by Ruiz-Bellido et al. [1].

The Functional Data Analysis was achieved using the R routines and “fda” functions for R software developed by Bi and Keusten [2] and Ramsay et al. [3]. Therefore, those interested in its application are kindly referred to their R routines and tutorial. Please, consult also [1] for detailed information of how raw data were processed and analysed.

## Figures and Tables

**Table 1:** Changes in yeast population (log_10_ cfu/ml) through the storage/fermentation process of Aloreña table olives. CC, control of acidified cured olives; CI, highly acidified cured olives; CII, intermediate acidified cured olives; CT, control of traditional (cracked) olives; RT, traditional olives, cracked after 72 h of exposure to air.

**Time (days)**	**CC**		**CI**		**CII**		**CT**		**RT**	
	1st Repl.	2nd Repl.	1st Repl.	2nd Repl.	1st Repl.	2nd Repl.	1st Repl.	2nd Repl.	1st Repl.	2nd Repl.
1	3.45	5.48	1.78	nd^a^	1.60	nd^a^	3.03	3.03	3.20	3.20
15	5.06	5.11	3.62	5.26	5.19	5.55	5.87	5.58	5.84	4.08
38	4.95	4.94	4.70	4.97	5.13	4.66	5.02	4.99	5.42	5.15
52	4.73	5.13	5.00	5.12	4.99	5.18	3.30	3.90	4.34	5.18
80	4.36	5.24	4.68	4.46	4.01	4.35	4.06	3.20	3.95	4.48
137	5.58	5.41	5.70	5.73	4.90	5.30	6.02	5.48	5.15	4.20
250	4.79	5.85	5.06	4.81	4.90	4.75	5.39	4.45	4.70	4.92
380	3.82	3.74	2.20	1.78	4.62	2.93	5.10	4.25	3.78	4.62

Repl. stands for replicate.

a nd, not detected (<1.3 log_10_ cfu/ml).

**Table 2:** Changes in pH through the storage/fermentation process of Aloreña table olives. CC, control of acidified cured olives; CI, highly acidified cured olives; CII, intermediate acidified cured olives; CT, control of traditional (cracked) olives; RT, traditional olives, cracked after 72 h of exposure to air.

**Time (days)**	**CC**		**CI**		**CII**		**CT**		**RT**	
	1st Repl.	2nd Repl.	1st Repl.	2nd Repl.	1st Repl.	2nd Repl.	1st Repl.	2nd Repl.	1st Repl.	2nd Repl.
1	2.71	2.71	2.37	2.37	2.40	2.40	2.71	2.71	2.71	2.71
15	3.79	3.73	3.32	3.32	3.33	3.35	4.32	4.39	4.42	4.48
38	4.21	4.17	3.91	3.79	3.96	3.99	4.34	4.40	4.35	4.45
52	4.26	4.21	3.97	3.95	4.17	4.06	4.36	4.43	4.35	4.33
80	4.46	4.30	4.13	3.91	4.12	4.08	4.34	4.44	4.40	4.36
137	4.41	4.15	4.22	4.13	4.21	4.17	4.38	4.35	4.32	4.31
250	4.62	4.25	4.28	4.08	4.28	4.23	4.30	4.30	4.31	4.36
380	4.43	4.00	4.08	4.01	4.14	4.09	4.55	4.21	4.20	4.24

Repl. stands for replicate.

**Table 3:** Changes in titratable acidity (g lactic/100 ml brine) through the storage/fermentation process of Aloreña table olives. CC, control of acidified cured olives; CI, highly acidified cured olives; CII, intermediate acidified cured olives; CT, control of traditional (cracked) olives; RT, traditional olives, cracked after 72 h of exposure to air.

**Time (days)**	**CC**		**CI**		**CII**		**CT**		**RT**	
	1st Repl.	2nd Repl.	1st Repl.	2nd Repl.	1st Repl.	2nd Repl.	1st Repl.	2nd Repl.	1st Repl.	2nd Repl.
1	0.61	0.61	2.40	2.40	1.60	1.60	0.61	0.61	0.61	0.61
15	0.49	0.49	1.59	1.87	1.26	1.33	0.49	0.49	0.44	0.44
38	0.37	0.38	1.53	1.50	1.20	1.18	0.40	0.40	0.4	0.31
52	0.43	0.43	1.55	1.48	1.12	1.14	0.43	0.43	0.41	0.41
80	0.49	0.47	1.51	1.37	1.06	1.12	0.39	0.41	0.39	0.34
137	0.77	0.81	1.91	1.92	1.68	1.59	0.54	0.53	0.54	0.54
250	0.44	0.71	1.75	2.02	1.47	1.55	0.46	0.50	0.44	0.38
380	0.94	1.11	2.36	2.45	1.89	2.01	0.43	0.59	0.53	0.48

Repl. stands for replicate.

**Table 4:** Average areas (±SE) below the yeast, pH and titratable acidity curves, according to treatments. CC, control of acidified cured olives; CI, highly acidified cured olives; CII, intermediate acidified cured olives; CT, control of traditional (cracked) olives; RT, traditional olives, cracked after 72 h of exposure to air.

**Treatment**	**Yeast**	**pH**	**Titratable acidity**
CC	1808 (64)	1618 (37)	253 (16)
CI	1505 (29)	1529 (17)	718 (13)
CII	1882 (76)	1553 (6)	576 (5)
CT	1820 (99)	1637 (6)	184 (5)
RT	1726 (34)	1627 (3)	171 (5)

Notes: One way ANOVA for the areas below the curves led to following *p*-values: 0.056, 0.003, and <0.001, for yeast, pH and titratable acidity, respectively.

**Table 5:** Changes in pH during storage/fermentation process of Aloreña table olives. CC, control of acidified cured olives; CI, highly acidified cured olives; CII, intermediate acidified cured olives; CT, control of traditional (cracked) olives; RT, traditional olives, cracked after 72 h of exposure to air. Parameters (±SE) of the model fit over time (*y*=*a*+*b*(1−exp(−cx))).

**Treatment**	***a***	***b***	***C* (days**^−^^**1**^**)**
CC	2.6±0.1	1.8±0.1	(8.6±1.5)E-2
CI	2.2±0.1	1.9±0.1	(6.6±0.7)E-2
CII	2.2±0.1	2.0±0.1	(6.9±0.7)E-2
CT	2.0±0.9	2.4±0.9	(0.35±0.38)⁎
RT⁎	--------⁎	--------⁎	--------⁎

*a*, intercept; *b*, overall change in pH; *c*, rate of pH change.

⁎ Non-significant parameters.

**Table 6:** Pairwise comparison of pH values between the areas of the different storage/fermentation Aloreña table olive treatments (Fisher LSD method, ANOVA *p*-value=0.003). CC, control of acidified cured olives; CI, highly acidified cured olives; CII, intermediate acidified cured olives; CT, control of traditional (cracked) olives; RT, traditional olives, cracked after 72 h of exposure to air.

**Comparison**	**Diff of Means**	**LSD** (**alpha=0.050**)	***P***	**Diff≥LSD**
CT vs. CI	108	68	0.009	Yes
CT vs. CII	84	68	0.024	Yes
CT vs. CC	20	68	0.489	No
CT vs. RT	10	68	0.728	No
RT vs. CI	99	68	0.013	Yes
RT vs. CII	74	68	0.037	Yes
RT vs. CC	10	68	0.721	No
CC vs. CI	89	68	0.020	Yes
CC vs.CII	64	68	0.059	No
CII vs. CI	25	68	0.396	No

**Table 7:** Pairwise comparison of titratable acidity values between the areas of the different storage/fermentation Aloreña table olive treatments (Fisher LSD method, ANOVA *p*-value<0.001). CC, control of acidified cured olives; CI, highly acidified cured olives; CII, intermediate acidified cured olives; CT, control of traditional (cracked) olives; RT, traditional olives, cracked after 72 h of exposure to air.

**Comparison**	**Diff of Means**	**LSD (α=0.050)**	***P***	**Diff≥LSD**
CI vs. RT	547	40	<0.001	Yes
CI vs. CT	534	40	<0.001	Yes
CI vs. CC	465	40	<0.001	Yes
CI vs. CII	142	40	<0.001	Yes
CII vs. RT	405	40	<0.001	Yes
CII vs. CT	393	40	<0.001	Yes
CII vs. CC	323	40	<0.001	Yes
CC vs. RT	82	40	0.002	Yes
CC vs. CT	70	40	0.005	Yes
CT vs. RT	12	40	0.427	No
