# Supplementary material for: Data on the application of Functional Data Analysis in food fermentations
Source: Data Brief. 2016 Sep 15;9:401–12. doi: 10.1016/j.dib.2016.09.013 (PMC5035237; doi:10.1016/j.dib.2016.09.013)
Supplement: Supplementary file 1 — Supplementary material [file mmc1.docx]

*Conflict of interest*

The authors declare that there is no conflict of interest.
